# Exploring the roles of different DNA Repair Proteins in Short Inverted Repeat Mediated Genomic Instability: A Pilot Study

**DOI:** 10.3390/dna4020008

**Published:** 2024-04-05

**Authors:** Pooja Mandke, Karen M. Vasquez

**Affiliations:** 1 Dell Pediatric Research Institute, Division of Pharmacology and Toxicology, College of Pharmacy, The University of Texas at Austin, 1400 Barbara Jordan Boulevard, Austin, TX 78723, USA

**Keywords:** Genomic instability, non-B DNA, DNA repair, ERCC1-XPF, NER, MMR, DSBR

## Abstract

Repetitive DNA sequences are abundant in the human genome and can adopt alternative (i.e., non-B) DNA structures. These sequences contribute to diverse biological functions, including genomic instability. Previously, we found that Z-DNA-, H-DNA-, and cruciform DNA-forming sequences are mutagenic, implicating them in cancer etiology. These sequences can stimulate the formation of DNA double-strand breaks (DSBs), causing deletions via cleavage by the endonuclease ERCC1-XPF. Interestingly, the activity of ERCC1-XPF in H-DNA-induced mutagenesis is nucleotide excision repair (NER)-dependent, but its role in Z-DNA-induced mutagenesis is NER-independent. Instead, Z-DNA is processed by ERCC1-XPF in a mechanism dependent on the mismatch repair (MMR) complex, MSH2-MSH3. These observations indicate distinct mechanisms of non-B-induced genomic instability. However, the roles of NER and MMR proteins and additional nucleases (CtIP and MRE11) in the processing of cruciform DNA remain unknown. Here, we present data on the processing of cruciform-forming short-perfect inverted repeats (IRs) by DNA repair proteins using mammalian cell-based systems. From this pilot study, we show that in contrast to H-DNA and Z-DNA, short perfect IRs are processed in a NER- and MMR-independent manner, and the nucleases CtIP and MRE11 suppress short perfect IR-induced genomic instability in mammalian cells.

## 1. Introduction

**Figure 1:**
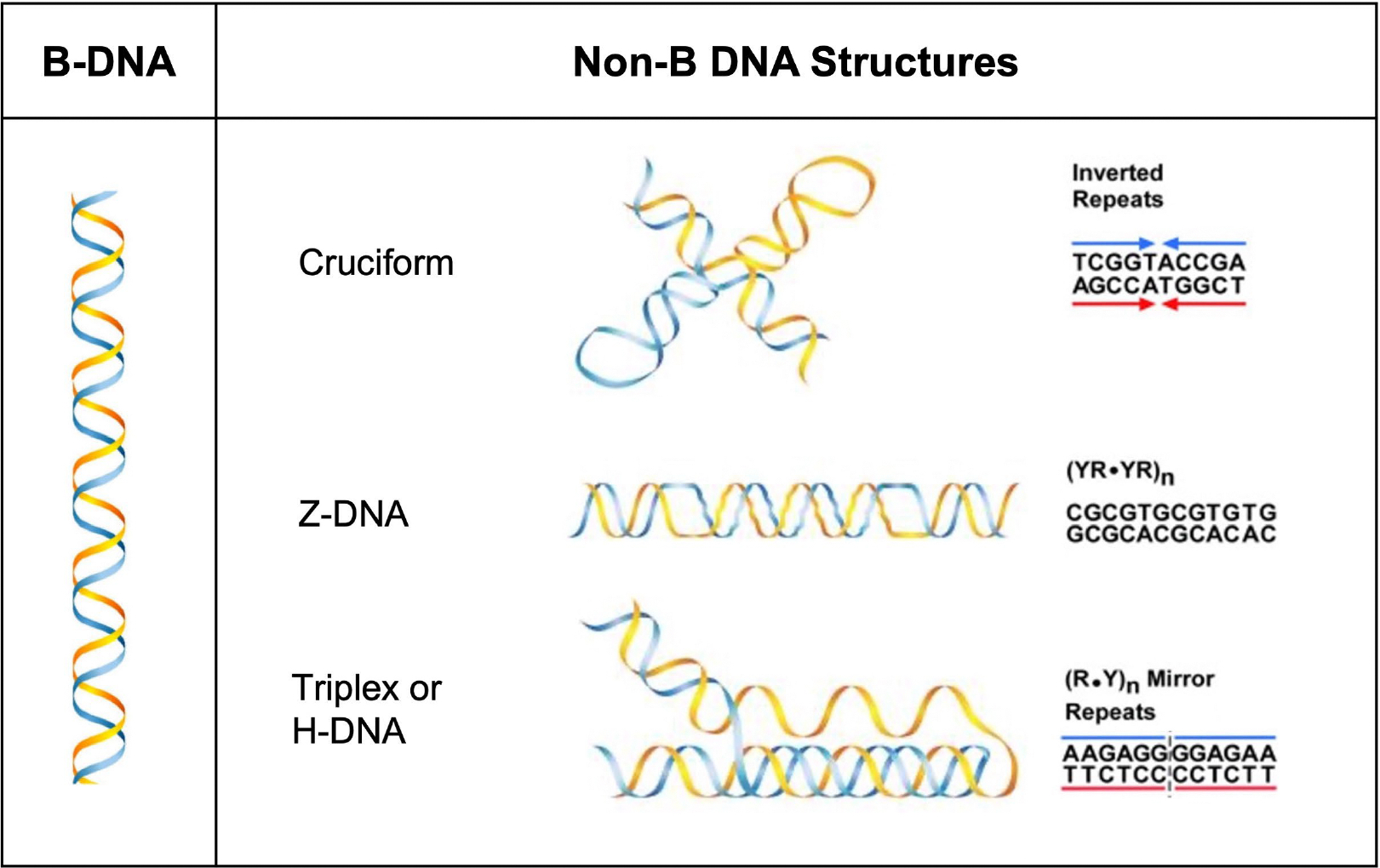
Schematic of non-B DNA structures. Left panel: canonical B-DNA duplex. Right panel: non-B DNA structures with the characteristic repetitive motifs at which they form. Listed here are cruciform DNA (inverted repeats), Z-DNA [alternating purines and pyrimidines; (YR-YR)n], triplex or H-DNA [mirror repeats; (R.Y)n]. (Adapted from 20)

Since the 1950s, over a dozen alternative or non-B DNA structures have been identified [1–4]. These structure-forming sequences have documented roles in physiological processes such as transcription, translation, replication, chromatin structure, and evolution; however, they also play roles in pathophysiological processes such as genomic instability, which can contribute to disease etiology [2,5–11]. Non-B DNA-forming sequences are abundant in the human genome, are markedly enriched at ‘hotspots’ of genomic instability in human cancer, and have been shown to be associated with human disease [12–16]. An overrepresentation of such sequences in frequently mutated loci also suggests that they are determinants of mutagenesis [10]. Hence, the abundance of repetitive DNA sequences that can adopt non-B DNA structures and their roles in normal physiological and pathophysiological conditions warrants further study of the mechanisms underlying their function and mutagenic processing. In this pilot study, we focus on determining the proteins involved in the mutagenic processing of cruciform DNA relative to our published mechanisms on the mutagenic processing of Z-DNA and H-DNA (FIGURE 1).

We have shown that Z-DNA-forming sequences induce large deletions and complex rearrangements in yeast, mammalian cells, and mouse chromosomes via the stimulation of DNA double-strand break (DSB) formation. This Z-DNA-induced genomic instability is facilitated by the endonuclease ERCC1-XPF, the recruitment of which is dependent on the mismatch repair (MMR) protein complex, MSH2-MSH3.

In addition, we have shown that H-DNA-forming sequences from the human *c-MYC* promoter can also stimulate the formation of DSBs, leading to large deletions in yeast, mammalian cells, and mouse chromosomes. Microhomologies at the deletion breakpoints indicate microhomology-mediated end-joining (MMEJ) mechanisms in the formation of the deletions [17–19]. Interestingly, we have identified both replication-dependent and replication-independent mechanisms of H-DNA-induced genomic instability. We have shown that ERCC1-XPF and XPG process H-DNA-forming sequences in a nucleotide excision repair (NER)-dependent manner, leading to large deletions via the formation of DSBs. This endonuclease-based cleavage mechanism could explain the replication-independent mechanisms of H-DNA-induced genomic instability [17]. In addition, we found that the absence of the flap endonuclease 1 (FEN1) protein led to an increase in H-DNA-induced mutagenesis, suggesting that FEN1 protects the genome from H-DNA obstructions during replication by cleaving this mutagenic structure.

With regard to inverted repeats (IRs), much of the research has focused on long IRs (>100 bp), which can stimulate deletion events in prokaryotes and yeast [20,21]. Similarly, long IRs have been studied extensively with regard to their contribution to human disease. However, a study of ~20,000 translocation breakpoints in human cancer genomes revealed IRs with stem lengths between 10–30 bps (short IRs) to be enriched within a 200 bp region around the breakpoints [15,22], implicating them in cancer etiology. We have also shown that a *short, perfect IR sequence* of 29 bp in length can stimulate DSBs, leading to deletions containing microhomologies at the breakpoint junctions in yeast and mammalian cells [22]. The DSBs can occur in a replication-independent manner via cleavage of the cruciform structures by the enzyme ERCC1-XPF or in a replication-dependent manner via fork stalling [22].

Hence, Z-DNA, H-DNA, and hairpin/cruciform DNA are biologically functional motifs and intrinsic sources of genomic instability in different organisms and cell types. We have found that all these sequences/structures are substrates for the enzyme complex ERCC1-XPF [17,22–24]. Interestingly, the role of ERCC1-XPF in H-DNA-induced genomic instability is NER dependent, while that associated with Z-DNA-induced mutagenesis is NER independent (but dependent on MSH2-MSH3). However, the roles of NER proteins on hairpins have shown conflicting results in both bacterial and mammalian cells, as reported by different groups, where their effects on CAG repeats resulted in either an increase or a decrease in deletion events, [25–27]. Additionally, MMR proteins can also bind/remove mismatch-containing small cruciform/hairpins, though not those formed by perfect IRs [28,29]. However, the role of MMR proteins in CAG repeat-mediated instability has shown conflicting results in mice and human systems, wherein the presence of MMR proteins, contraction events at CAG repeats were seen in humans. [26], while expansion events were seen in mice [30].

We have shown that non-B DNA structures induce genomic instability via the stimulation of DSB formation, resulting in large deletions [17,22,23]. These large deletions show microhomologies at the breakpoint junctions, suggesting a role of MMEJ in processing the DSBs induced by non-B DNA structures. An important step involved in the initiation of this pathway is DNA end resection at the site of a DSB catalyzed by the CtIP and MRE11 proteins [31]. Interestingly, apart from their roles in homologous recombination (HR), MRE11 and CtIP have also been shown to mediate MMEJ repair mechanisms [32]. In addition to its role in resection, MRE11 can also function as an endonuclease and cleave hairpin loops *in vitro* [33–35]. Interestingly, other groups have demonstrated endonuclease-mediated cleavage of single-stranded DNA next to the hairpins by the yeast homolog of CtIP, i.e., Sae2 [35,36]. While the CtIP protein augments the function of MRE11, the studies on its endonuclease functions are inconsistent [37]. Relevant to non-B DNA processing, CtIP and MRE11 are required to preserve stability at AT-rich common fragile sites and Alu-IRs [38], demonstrating that CtIP and MRE11 could have diverse roles in processing structures formed at IRs. In addition, the small loop within the cruciform structure may also provide a substrate for CtIP and MRE11. Hence, while CtIP and MRE11 may process IR-induced DSBs via HR or MMEJ, they could also be involved in cleaving the structures formed at IRs.

Together, these studies indicate that while ERCC1-XPF can cleave a variety of non-B DNA structures, distinct DNA repair mechanisms/proteins are involved in their mutagenic processing. Since the mechanisms involved in the mutagenic processing of short-perfect IRs are not clear, we performed a preliminary analysis of different DNA repair proteins and their roles in short-perfect IR-induced genomic instability in human cells. Thus, in this pilot study, we sought to ascertain the involvement of NER and MMR proteins and additional DNA repair proteins (CtIP and MRE11) on short-perfect IR-induced genomic instability.

## 2. Materials and Methods

### 2.1. Mutagenesis assays in mammalian cell lines

We utilized mutation-reporter shuttle vectors to study non-B DNA-induced mutagenesis [39], which can replicate in both bacterial and mammalian cells. For this study, we used the pSP189-based mutation-reporters containing the SV40 origin of replication and T antigen, which allows for replication in mammalian cells, along with the pBR327 origin of replication, which allows for replication in bacterial cells. This reporter also contains the *supF* mutation-reporter gene, which facilitates the use of bacterial strains carrying an amber mutation as indicators of functional activity of the *supF* gene via blue-white screening [39–41]. In this study we have cloned a 29-bp IR sequence capable of forming cruciform DNA structures (**IR)** into the *supF*-containing reporter vector, pSP189, using standard cloning protocols. The *supF*-containing pSupFG1 vector was used as a control (**B-DNA)** reporter.

### 2.2. Cell lines

Human XPA-deficient (XPA2OSSV) and XPA-proficient (SV40-immortalized human fibroblasts complemented with the XPA gene) [42,43] were maintained in Dulbecco’s Modified Eagle’s Medium with supplements [DMEM + 10% heat-inactivated fetal bovine serum (HI-FBS) + 1% Penicillin-Streptomycin]. Human XPF-deficient and XPF-proficient [44] cells were maintained in Dulbecco’s Modified Eagle’s Medium with supplements [DMEM + 10% fetal bovine serum (FBS) + 1% Penicillin-Streptomycin].

### 2.3. siRNA-mediated knockdown of DNA repair proteins and mutation reporter transfection

We performed siRNA-mediated transient knockdowns to study the effects of different proteins on IR-induced mutagenesis. Two transfections were done with the ON-TARGET plus siRNA SMARTpool for human (MSH2, CtIP, or MRE11) and non-targeting control SMARTpool. A *reverse transfection* was performed using 25–40 nM siRNA in OptiMEM Reduced-Serum Medium. The 48-hour time point was selected as it gave >80% knockdown of protein levels at the time of mutation reporter transfection into the human cells. Next, a *forward transfection* of siRNA was performed with 2.5 μg of the IR- or B-DNA-containing reporters with the GenePORTER transfection reagent (Genlantis Inc, now AMSBIO, San Diego, CA). Samples were collected at T_48_ and T_96_ (48 hours after the second transfection) to verify protein knockdown by western blotting using the following antibodies; anti-MSH2 (Cell-Signaling Technology), anti-CtIP (Cell Signaling Technology) and anti-MRE11 (Novus Biologicals). Transfected reporters were collected at T_96_ by the alkaline lysis method using QIAPrep Spin Miniprep kit (Qiagen, Germantown, MD) with slight modifications to the protocol. The extracted mutation reporters were DpnI digested, purified and then used for the transformation of electrocompetent MBM7070 cells to determine mutation frequency via blue-white screening.

### 2.4. Mutation frequency and mutation spectra analyses

Mutation frequencies were determined by dividing the total number of white (mutant) colonies by the total number of blue (wild-type), and white (mutant) colonies counted on X-Gal, IPTG, and carbenicillin agar plates. Experiments were performed in triplicate, and at least 20,000 colonies were counted for each replicate. Statistical significance was calculated using a two-way ANOVA test.

Mutation spectra were evaluated by sequencing randomly selected white mutant colonies and a control blue wild-type colony. Mutation reporter isolation was performed using the QIAPrep Spin Miniprep kit. The isolated reporters were sequenced by Sanger’s sequencing method using primers specific for a region around the mutation-reporter gene and the IR or control B-DNA sequence (seqPriim189 CAAAAAAGGGAATAAGGGCG). The obtained sequences were used to characterize the types of mutations around the IR sequence and a similar region in the control B-DNA reporter.

## 3. Results And Discussion

Based on substrate preferences, short perfect IRs may be recognized and processed by NER, MMR, MMEJ, and/or HR repair proteins/pathways. Studies to determine which proteins are involved are ongoing, and the initial findings are presented below.

**Figure 2.**
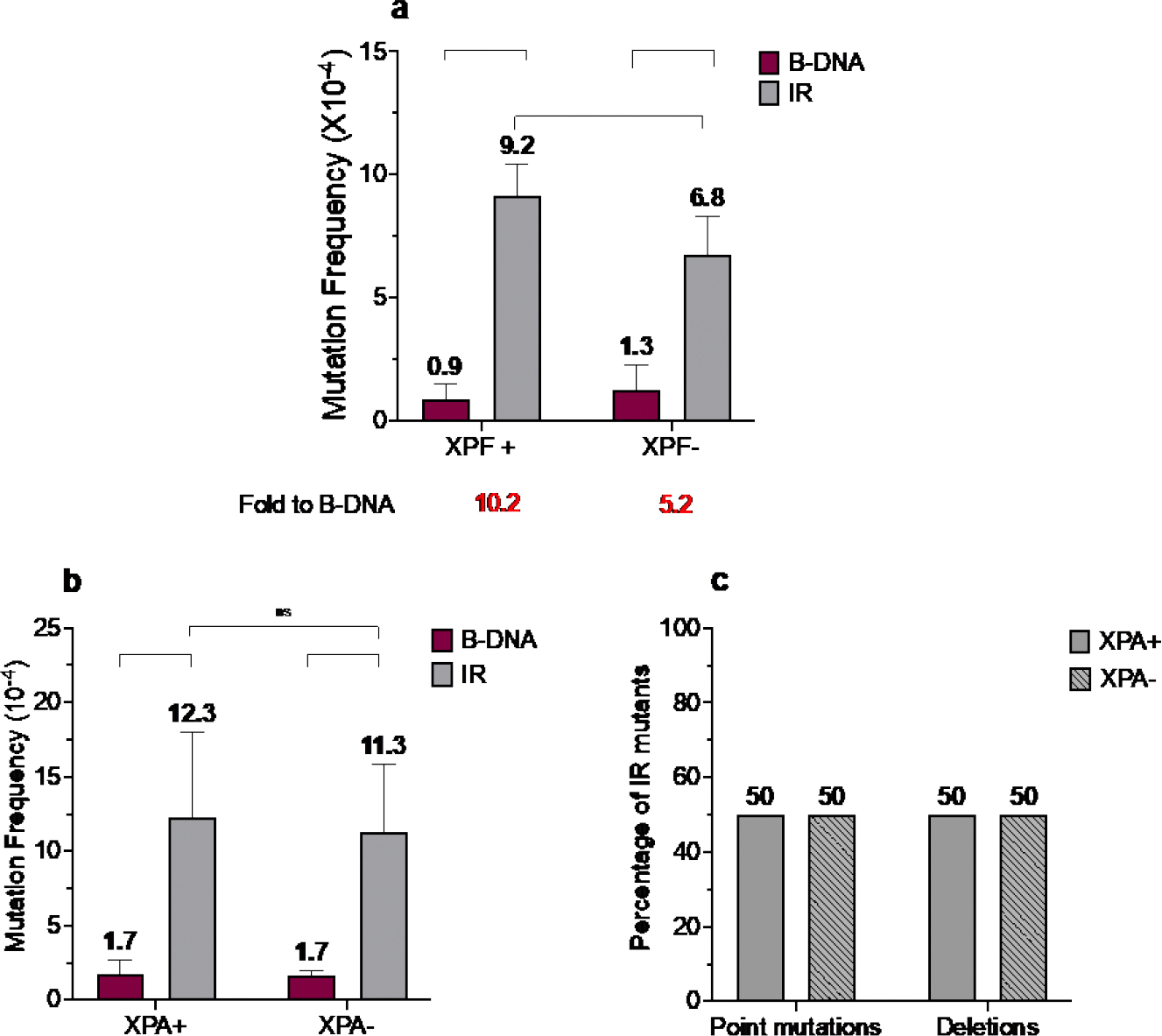
IR-induced mutagenesis decreases in the absence of ERCC1-XPF and remains unchanged in the presence or absence of XPA. a) Mutation frequencies were measured in human XPF-proficient fibroblast cell lines in the presence of control siRNA. Mutation reporters containing control (B-DNA) sequences or short, perfect inverted repeat (IR) sequences were transfected into the human XPF-proficient cells at T_48_ and collected 48 hours later at T_96_. Mutation frequencies were calculated as the ratio of white colonies to the total number of colonies. Experiments were performed in triplicate; data are expressed as mean ± SD and 2-way ANOVA with Šidák post hoc test, *p<0.05, **p<0.01, ***p<0.001, ****p<0.0001 was used for statistical analysis. b) Mutation frequencies were measured in human XPA-proficient and deficient cell lines. Mutation reporters containing control (B-DNA) sequences or short perfect inverted repeat (IR) sequences were transfected into the cells and isolated 48 hours post-transfection. Mutation frequencies were calculated as the ratio of white (mutant) colonies to the total number of colonies (blue plus white). Experiments were performed in triplicate. Data are expressed as mean ± SD and 2-way ANOVA with Šidák posthoc test, *p<0.05, **p<0.01, ***p<0.001, ****p<0.0001 was used for statistical analysis. c) Percentage distribution of different types of mutants. Mutants are characterized as point mutations and deletions. Grey solid bars represent samples from XPA-proficient cells, and grey patterned bars represent samples from XPA-proficient cells.

It is known that NER is required for the repair of helix-distorting bulky adducts [45,46], thereby validating its potential role in processing non-B DNA structures as seen with the mutagenic processing of H-DNA [17]. We have previously shown that ERCC1-XPF (a key nuclease in the NER pathway) is involved in IR-induced genomic instability in yeast and mammalian systems [22]. In line with our previous observation in mammalian cells, we found that short perfect IRs are mutagenic in human cells, with a 10.2-fold increase in IR-induced mutagenesis over that of control B-DNA in wild-type human XPF-proficient fibroblasts (FIGURE 2a). Further, ERCC1-XPF plays a role in short-perfect IR-induced mutagenesis in human cells, as seen by the attenuation (5.2-fold increase) in IR-induced mutagenesis over that of control B-DNA in the human XPF-deficient fibroblasts accompanied by a decrease in mutation frequency in the absence of XPF (9.2 X10^−4^ *vs.* 6.8 X 10^−4^, p<0.1) (FIGURE 2a, *grey bars*). Interestingly, while short perfect IRs induced mutations in both the presence and absence of XPA (a critical NER recognition and verification protein), the presence (12.3 X 10^−4^) or absence of XPA (11.3 X 10^−4^) did not impact the short perfect IR-induced mutagenesis (FIGURE 2b, *grey bars*).

We did not detect any significant differences in the mutation spectra associated with the IR-induced genomic instability in human XPA-proficient *vs.* deficient cells, with ~50% deletions and ~50% point mutations observed in both cell lines (FIGURE 2c). Thus, it appears that unlike H-DNA [17], functional NER is not required for the mutagenic processing of short-perfect IRs in human cells.

**Figure 3.**
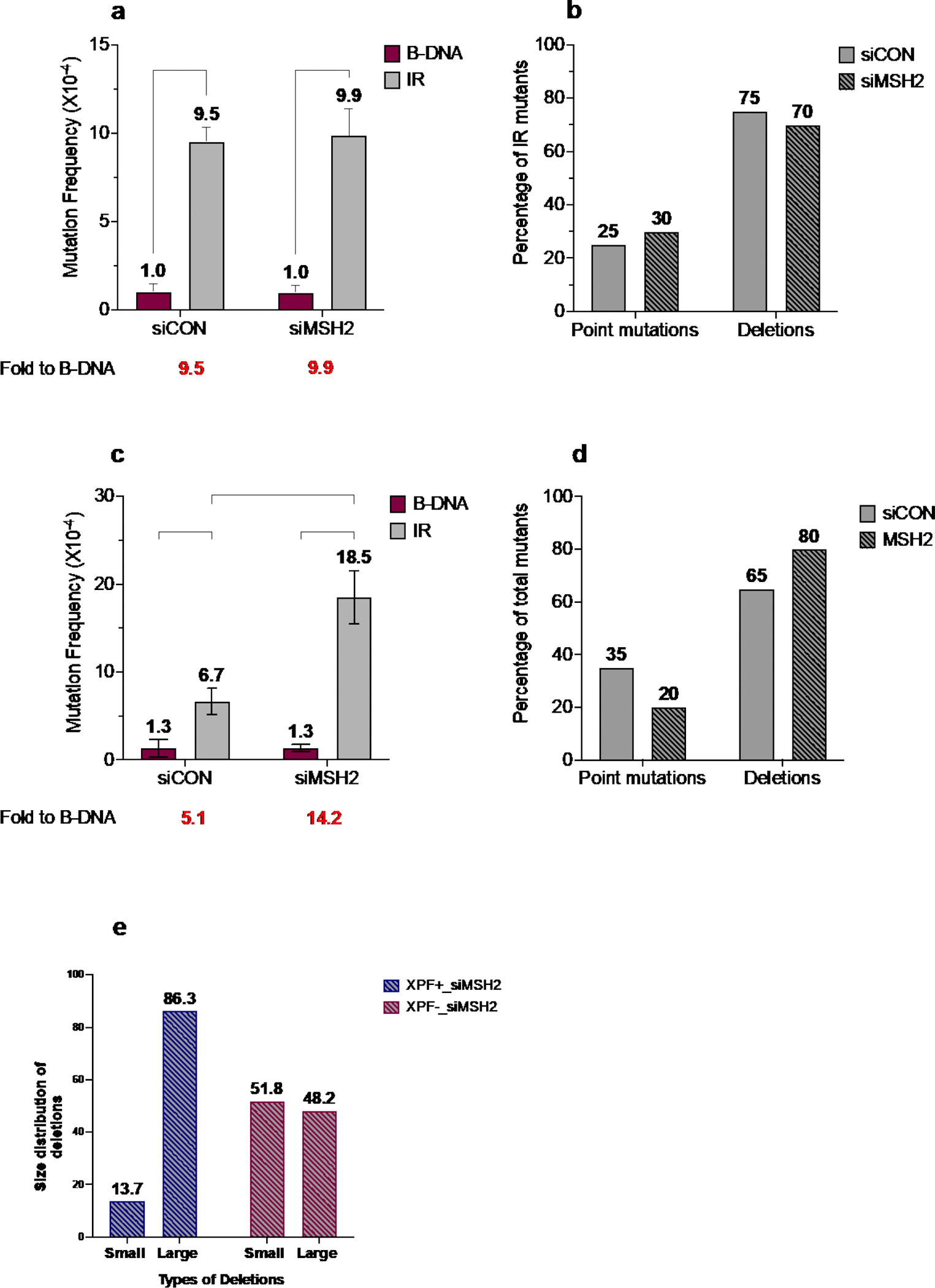
IR-induced mutagenesis remains unchanged in the absence of MSH2 and increases in the absence of both MSH2 and XPF. a) Mutation frequencies were measured in human XPF-proficient cell lines in the presence of siCON or siMSH2. Mutation reporters containing control (B-DNA) sequences or short perfect inverted repeat (IR) sequences were transfected into the human XPF-proficient cells at T_48_ and collected 48 hours later at T_96_. Mutation frequencies were calculated as the ratio of white colonies to the total number (blue plus white) of colonies counted. Experiments were performed in triplicate; data are expressed as mean ± SD and 2-way ANOVA with Šidák posthoc test, *p<0.05, **p<0.01, ***p<0.001, ****p<0.0001 was used for statistical analysis. b) Percentage distribution of different types of mutants in human XPF-proficient cell lines following treatment with siCON or siMSH2. Mutants are characterized as point mutations and deletions. Grey solid bars represent siCON-treated samples, and grey patterned bars represent siMSH2-treated samples. c) Mutation frequencies were measured in human XPF-deficient cell lines in the presence of siCON or siMSH2. Mutation reporters containing control (B-DNA) sequences or short perfect inverted repeat (IR) sequences were transfected into the human XPF-deficient cells at T_48_ and collected 48 hours later at T_96_. Mutation frequencies were calculated as the ratio of white colonies to the total number (blue plus white) of colonies counted. Experiments were performed in triplicate; data are expressed as mean ± SD and 2-way ANOVA with Šidák posthoc test, *p<0.05, **p<0.01, ***p<0.001, ****p<0.0001 was used for statistical analysis. d) Percentage distribution of different types of mutants in human XPF-deficient cell lines following treatment with siCON or siMSH2. Mutants are characterized as point mutations and deletions. Grey solid bars represent siCON-treated samples, and grey patterned bars represent siMSH2-treated samples. e) Comparative analysis of short perfect IR-induced deletions from human XPF-proficient and XPF-deficient cells treated with siMSH2. The deletions were categorized as small deletions (<100 bp) and large deletions (>100 bp). Blue patterned bars correspond to XPF-proficient cells treated with siMSH2, and red patterned bars represent human XPF-deficient cells treated with siMSH2.

Since we have previously shown that the cleavage of Z-DNA by ERCC1-XPF was dependent on its interaction with MSH2-MSH3, we evaluated the effect of MSH2 (an essential MMR protein) in the presence or absence of XPF on short-perfect IR-induced genomic instability. We found that short perfect IR-induced genomic instability was not different in the presence (9.5 X 10^−4^) or absence (9.9 X 10^−4^) of MSH2 (FIGURE 3a, *grey bars*). This is not unexpected, given previous reports indicating that MMR proteins bind more specifically to mismatch-containing hairpins/cruciforms rather than to perfect repeats [29]. In support of this, we also did not observe an association of MSH2 with the short-perfect IR sequences in our plasmid-based ChIP assays (data not shown). Thus, it appears that unlike Z-DNA [23], MSH2 is not required for the mutagenic processing of short perfect IRs in human cells.

Upon analysis of the mutation spectra, we did not observe any considerable differences in the distribution of mutation types associated with the IR-induced genomic instability in the presence or absence of MSH2, with ~25% point mutations and ~75% deletions observed in mutants from both the control and MSH2-depleted cells (FIGURE 3b). However, a difference was seen in the position of the breakpoint junctions around the IR sequence in the presence of MSH2 compared to the those detected in the absence of MSH2. In the presence of MSH2, most of the breakpoint junctions were located in the tip of the loop region *within* the IR sequence, resulting in *partial loss* of the IR sequence. In contrast, in the absence of MSH2, ~50% of the breakpoint junctions were mapped *outside* the IR sequence and resulted in the *complete loss* of the IR sequence (data not shown).

MSH2 has varied functions in several DNA repair pathways, such as MMR, HR, single-strand annealing (SSA), etc. [47–50], and interacts with and stimulates or inhibits the activity of different proteins [48]. For example, MutSβ (MSH2-MSH3) can stimulate the activity of the SMX trinuclease in Holliday junction (HJ) resolution [51]. MutSα (MSH2-MSH6) can interact with the BLM helicase and promote dissolution of HJs [52]. Because Holliday junctions are structurally similar to cruciform DNA, we speculate that MSH2, via its interacting partners MSH3, MSH6, or other interacting proteins, may regulate the processing of structures formed at short-perfect IRs. Hence, the presence or absence of MSH2 could lead to differential processing around the IR sequence, leading to alterations in the mutation spectra.

Surprisingly, we observed that the absence of both MSH2 and XPF led to an increase in IR-induced genomic instability (6.7 X 10^−4^ *vs.* 18.5 X 10^−4^) (FIGURE 3c, *grey bars*). We observed a minor shift in the mutation spectra associated with IR-induced genomic instability in the presence or absence of MSH2, with ~65% deletions and ~35% point mutations in mutants from the wild-type cells, and ~80% deletions and ~20% point mutations in mutants from the MSH2-depleted cells (FIGURE 3d). Interestingly, we also observed an alteration in the size of the deletions in MSH2-depleted samples in the presence or absence of XPF. The deletions were grouped as >100 bp or <100 bp; in the presence of XPF, ~14% of deletions were <100 bp and the remaining ~86% were >100 bp (*blue dashed bars*). In contrast, ~52% of deletions were <100 bp and the remaining ~48% were >100 bp in the absence of XPF (*red dashed bars)* (FIGURE 3e). Mutants from the control B-DNA samples predominantly contained point mutations in the presence or absence of MSH2.

The differences in the sizes of the deletions generated in the human XPF-proficient or deficient cells in the absence of MSH2 could be attributed to the different mechanisms that might be involved in processing the short IRs as a result of the interplay between ERCC1-XPF and MSH2. Hence, under these conditions, one might speculate on the roles of additional proteins/nucleases in the mutagenic processing of short IRs. Because IRs can adopt cruciform structures, they may be susceptible to processing by enzymes that cleave around the single-stranded regions (loops), as seen with ERCC1-XPF-mediated cleavage of the 29-bp IR substrate around the loop region indicating a ‘*center break*’ mechanism [22,53]. In addition, the presence of four-way junctions in the cruciform structure, which are remarkably similar to an Holliday junction intermediate, could make these potential substrates for HJ resolvases (e.g., GEN1, SLX1, MUS81) [54–56] in a ‘*resolution’*-type mechanism. Interestingly, Inagaki et al. (2013) have shown that palindrome-mediated translocations associated with the PATRR entail two sequential cleavage reactions by the GEN1 and Artemis proteins, suggesting a mechanism that involves resolution of the four-way junction of the cruciform structure [57]. Additionally, Kaushal et al. (2019) have shown that the fragility linked to the Flex1 long AT-rich region of the common fragile site FRA16D could be attributed to its capacity to adopt a cruciform structure. The fragility involves the yeast Mus81-Mms4 and Slx1–4/Rad1–10 protein complexes [58]. Since these proteins play a role in Holliday junction resolution and processing of cruciform structures formed at long IRs, additional studies must be undertaken to determine their roles and those of other proteins in short perfect IR-induced genomic instability.

**Figure 4.**
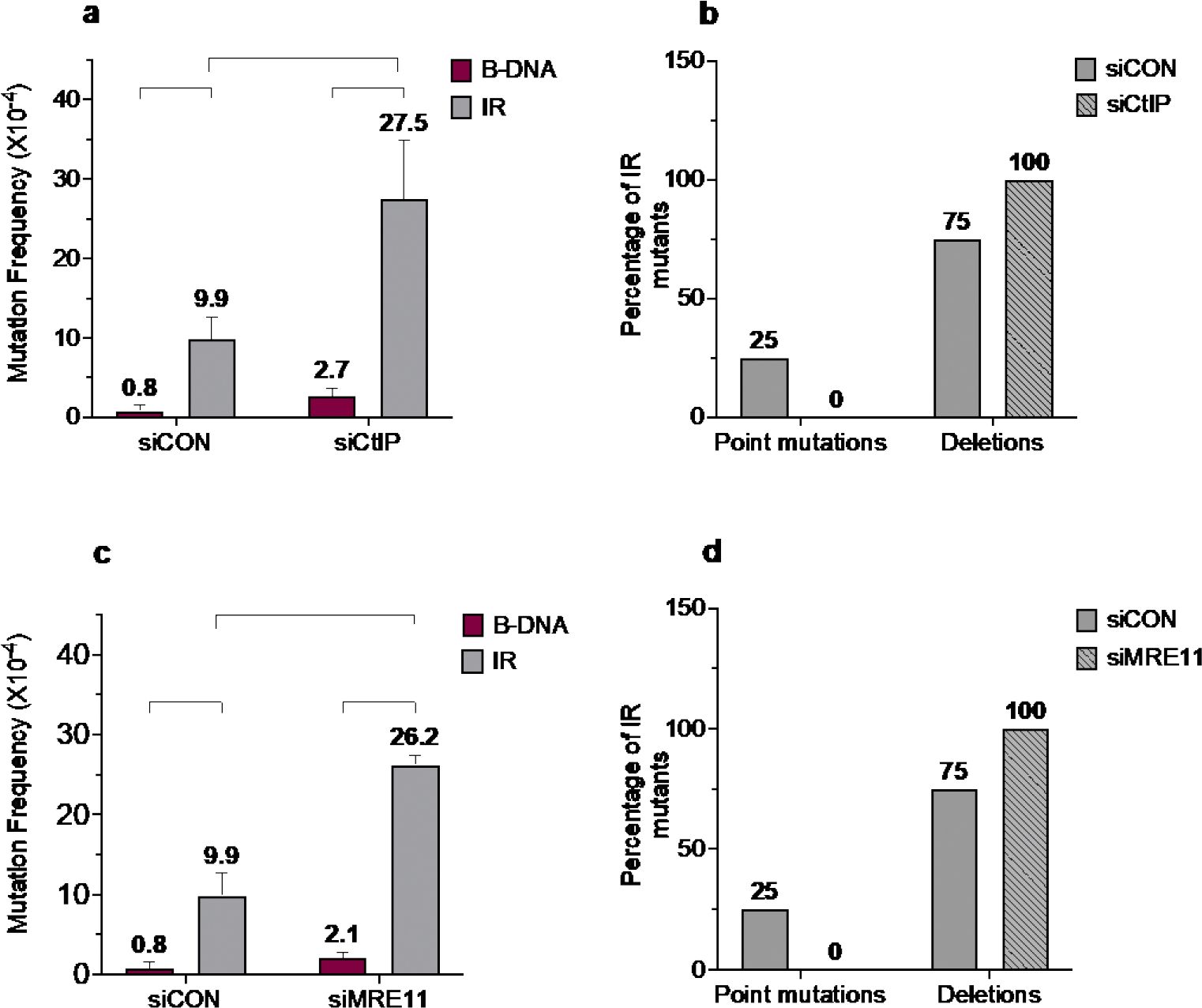
IR-induced mutagenesis increases in the absence of CtIP and MRE11. a) Mutation frequencies were measured in human XPF-proficient cell lines in the presence of siCON or siCtIP. Mutation reporters containing control (B-DNA) sequences or short perfect inverted repeat (IR) sequences were transfected into the human XPF-proficient cells at T_48_ and collected 48 hours later at T_96_. Mutation frequencies were calculated as the ratio of white colonies to the total number of colonies. Experiments were performed in triplicate; data are expressed as mean ± SD and 2-way ANOVA with Šidák post hoc test, *p<0.05, **p<0.01, ***p<0.001, ****p<0.0001 was used for statistical analysis. b) Percentage distribution of different types of mutants in human XPF-proficient cells following treatment with siCON or siCtIP. Mutants are characterized as point mutations and deletions. Grey solid bars represent siCON-treated samples, and grey patterned bars represent siCtIP-treated samples. c) Mutation frequencies were measured in human XPF-proficient cell lines in the presence of siCON or siMRE11. Mutation reporters containing control (B-DNA) sequences or short perfect inverted repeat (IR) sequences were transfected into the human XPF-proficient cells at T_48_ and collected 48 hours later at T_96_. Mutation frequencies were calculated as the ratio of white colonies to the total number of colonies. Experiments were performed in triplicate; data are expressed as mean ± SD and 2-way ANOVA with Šidák post hoc test, *p<0.05, **p<0.01, ***p<0.001, ****p<0.0001 was used for statistical analysis. d) Percentage distribution of different types of mutants in human XPF-proficient cells following treatment with siCON or siMRE11. Mutants are characterized as point mutations and deletions. Grey solid bars represent siCON-treated samples, and grey patterned bars represent siMRE11-treated samples.

Previously, we demonstrated a role for the ERCC1-XPF nuclease in the mutagenic processing of short perfect IRs [22]. Here, we extended our study to include the CtIP and MRE11 nucleases (critical in DSB repair processing) [59,60]. We found that short perfect IR-induced genomic instability increased in the absence of CtIP (9.9 X 10^−4^ *vs.* 27.5 X10^−4^) and MRE11 (9.9 X 10^−4^ *vs.* 26.2 X10^−4^) in siRNA-depleted human fibroblasts (FIGURE 4a and 4c). This suggests a role for CtIP and MRE11 in *suppressing* IR-induced genomic instability. Upon analysis of the mutation spectra, we observed a shift in the mutation types between the wild-type cells and the CtIP- or MRE11-depleted cells. When CtIP or MRE11 were depleted, 100% of the mutants contained deletion events, whereas the wild-type cells had ~75% deletion events and ~25% point mutations (FIGURE 4b and 4d).

As mentioned previously, both CtIP and MRE11 have varied roles in the DNA damage repair processes; in this case, CtIP and MRE11 could favor the repair of the IR-induced DSBs by the error-free HR pathway. Hence, these proteins could potentially restore stability at IR-induced DSBs. Because these events are error-free, they will not be captured by our blue-white screening assay. However, the presence of IR-induced deletions in the absence of CtIP and MRE11 is intriguing. Since both CtIP and MRE11 are regulators of the key step of 5’−3’ resection, the depletion of these proteins could affect resection at the IR-induced DSBs. Since deletion events may require resection at the DSBs, it is possible that other nucleases are able to compensate, in part, for the resection function of MRE11. One such protein is EXO1, a 5’−3’ exonuclease involved in revealing long tracts of ssDNA after initial resection by MRE11/CtIP [61,62].

Interestingly, it has been shown that the yeast homologs of MRE11 and EXO1, i.e., Mre11 and Exo1, have overlapping functions, suggesting redundancy in the resection functions with defects in Mre11 being compensated for by overexpression of ExoI [63,64]. Studies in yeast have also found roles for ExoI in repair via MMEJ mechanisms [65]. Perhaps in the absence of CtIP/MRE11, EXO1 could, to a limited extent, facilitate resection at the short IR-induced DSBs and promote the more mutagenic MMEJ pathway, leading to deletions observed in the absence of CtIP and MRE11.

Our study provides evidence to suggest that short perfect IRs may be processed in a manner distinct from that of H-DNA- and Z-DNA-forming sequences, i.e., the mutagenic processing of short perfect IRs appears to be independent of a functional NER pathway and of MSH2, which is required for functional MMR.

## 4. Concluding Remarks

Previously, we demonstrated that the mutagenic processing of different non-B DNA structures involves distinct mechanisms, with H-DNA being processed by NER and Z-DNA being processed by the NER nuclease, ERCC1-XPF, which requires the MMR complex, MSH2-MSH3 for the recruitment of ERCC1-XPF to the Z-DNA region [17,23,66]. This study provides preliminary evidence of the roles of different DNA repair proteins in the mutagenic processing of cruciform-forming short perfect IRs. Due to the complex interplay between different DNA repair proteins on this structure, several protein/pathways are likely involved in its mutagenic processing. Future studies are necessary to define the exact mechanism(s) involved in IR-induced genomic instability. Understanding the mechanisms responsible for DNA structure-induced genomic instability and their roles in disease etiology will allow for the development of therapeutics to treat and/or prevent diseases of genetic instability, such as cancer and neurodegenerative disorders.

## Data Availability

Not applicable.
